# VRE and VSE Bacteremia Outcomes in the Era of Effective VRE Therapy: A Systematic Review and Meta-analysis

**DOI:** 10.1017/ice.2015.228

**Published:** 2015-10-05

**Authors:** Chatura Prematunge, Colin MacDougall, Jennie Johnstone, Kwaku Adomako, Freda Lam, Jennifer Robertson, Gary Garber

**Affiliations:** 1 Infection Prevention and Control, Public Health Ontario, Toronto, Ontario, Canada; 2 St. Joseph’s Health Centre, Toronto, Ontario, Canada; 3 Department of Medicine, University of Toronto, Toronto, Ontario, Canada; 4 Department of Medicine, University of Ottawa, Ottawa, Ontario, Canada; 5 Ottawa Hospital Research Institute, Ottawa, Ontario, Canada

## Abstract

**BACKGROUND:**

Prior data suggest that vancomycin-resistant *Enterococcus* (VRE) bacteremia is associated with worse outcomes than vancomycin-sensitive *Enterococcus* (VSE) bacteremia. However, many studies evaluating such outcomes were conducted prior to the availability of effective VRE therapies.

**OBJECTIVE:**

To systematically review VRE and VSE bacteremia outcomes among hospital patients in the era of effective VRE therapy.

**METHODS:**

Electronic databases and grey literature published between January 1997 and December 2014 were searched to identify all primary research studies comparing outcomes of VRE and VSE bacteremias among hospital patients, following the availability of effective VRE therapies. The primary outcome was all-cause, in-hospital mortality, while total hospital length of stay (LOS) was a secondary outcome. All meta-analyses were conducted in Review Manager 5.3 using random-effects, inverse variance modeling.

**RESULTS:**

Among all the studies reviewed, 12 cohort studies and 1 case control study met inclusion criteria. Similar study designs were combined in meta-analyses for mortality and LOS. VRE bacteremia was associated with increased mortality compared with VSE bacteremia among cohort studies (odds ratio [OR], 1.80; 95% confidence interval [CI], 1.38–2.35; I^2^=0%; n=11); the case-control study estimate was similar, but not significant (OR, 1.93; 95% CI, 0.97–3.82). LOS was greater for VRE bacteremia patients than for VSE bacteremia patients (mean difference, 5.01 days; 95% CI, 0.58–9.44]; I^2^=0%; n=5).

**CONCLUSIONS:**

Despite the availability of effective VRE therapy, VRE bacteremia remains associated with an increased risk of in-hospital mortality and LOS when compared to VSE bacteremia.

*Infect. Control Hosp. Epidemiol.* 2015;37(1):26–35

*Enterococcus* spp. are typically commensal organisms, common in the human gastrointestinal tract,^1^ ^,^ ^2^ but in some circumstances can cause serious infections including bacteremia, particularly among hospitalized patients with underlying comorbid conditions.^1^ ^,^ ^2^ Since its discovery in 1988, vancomycin-resistant enterococci (VRE) have emerged as important nosocomial pathogens and are occurring with increasing frequency due to widespread use of antibiotics, prolonged hospitalizations, and increased intensive care unit (ICU) admissions, especially among patients with malignant health conditions.^1^ ^–^ ^3^ In Canada, the incidence of VRE infections has risen to 0.5 infections per 1,000 admissions, a 6-fold increase in recent years.^4^ Similarly in the United States, hospitalizations with VRE infection reached 0.6 per 1,000 admissions by 2006.^3^

Whether outcomes associated with VRE bacteremia are worse than those associated with vancomycin-sensitive enterococci (VSE) bacteremia remains unclear. Two prior systematic reviews have compared outcomes of VRE bacteremia VSE bacteremia; both found VRE bacteremia to be associated with an increased risk of mortality when compared to VSE bacteremia (relative risk [RR], 2.38; 95% confidence interval [CI], 2.13–2.66;^5^ odds ratio [OR], 2.52; 95% CI, 1.87–3.39^6^). However, both of these systematic reviews included studies conducted prior to the availability of effective VRE therapies.^5^ ^,^ ^6^ Since late 1999, a number of antibiotic drugs have been licensed as treatment for VRE bacteremia by the US Food and Drug Administration (FDA), Health Canada, and other national approval agencies.^1^ ^,^ ^2^ ^,^ ^7^ Quinupristin-dalfopristin was approved in 1999, followed by linezolid in 2000.^7^ In 2003, daptomycin was formally licensed for complicated skin and soft tissue VRE infections, but it is frequently used as an off-label therapy for VRE bacteremia.^8^

Thus, understanding whether VRE bacteremia-associated outcomes are different from those of VSE bacteremia, since the emergence of effective VRE therapy, is critically important to help inform future VRE infection control recommendations. To this end, we performed a systematic review and meta-analysis of studies comparing outcomes of patients with either VRE or VSE bacteremia, when patients with VRE bacteremia were treated with effective VRE therapy.

## Methods

All methods including literature searches, study selection, data collection, and quantitative analysis processes were developed *a priori* and were reported according to the Preferred Reporting Items for Systematic Reviews and Meta-Analyses (PRISMA) guidelines and the *Cochrane Handbook for Systematic Reviews of Intervention*.^9^ ^,^ ^10^

### Search Methodology and Data Sources

The Public Health Ontario (PHO) Library Services department assisted with the development and implementation of search strategies for electronic databases, as well as with the retrieval of full-text articles. Medline, Embase, CINAHL, ProQuest dissertations and theses, and the Cochrane Central Register of Controlled Trials (CENTRAL) databases were searched from January 1997 to December 2014. A sample search strategy is provided in Supplemental Table 1. Websites of infection control authorities and proceedings from infection control conferences held within the most recent 5 years (ie, January 1, 2010, to January 1, 2015) were searched as outlined in Supplemental Table 2. Conference proceedings prior to 2010 were not considered because we assumed that valuable data contained within such abstracts had become available in peer-reviewed literature. Additionally, the reference lists of all relevant publications were hand searched to identify additional citations.

### Study Inclusion Criteria

The study inclusion criteria for the review were randomized controlled trials (RCTs), cohort studies, case-control studies, and cross-sectional studies, sampling adult (≥18 years of age) and/or pediatric (<18 years of age) hospital patients, diagnosed with VRE bacteremia and treated with effective VRE therapy, alongside VSE bacteremia patient comparators, and reporting on various mortality and morbidity outcomes. The primary outcome of interest was all-cause in-hospital mortality. Secondary outcomes were bacteremia-attributable mortality, total hospital length of stay (LOS), total intensive care unit (ICU) LOS, post-VRE/VSE bacteremia diagnosis hospital LOS, and post-VRE/VSE bacteremia diagnosis ICU LOS. Effective VRE therapies were defined as quinupristin-dalfopristin, linezolid, daptomycin, tigecycline, teicoplanin, and telavancin for treating any part of the illness.^1^ ^,^ ^2^ ^,^ ^7^ ^,^ ^8^ Penicillin, ampicillin, amikacin, streptomycin, chloramphenicol, doxycycline, rifampin, imipenem-cilastatin, and nitrofurantoin were not considered effective VRE treatments.^11^

To capture standard, off-label, and compassionate study use of effective VRE treatment(s), literature published after January 1997 was considered. Studies analyzing data collected between January 1997 and January 2000 were excluded if the antibiotics used for the treatment of VRE bacteremia patients were not reported or could not be obtained by contacting study authors. Studies conducted after January 2000 were assumed to have administered effective VRE treatment(s) and were included in the review.

Narrative reviews, case series, case reports, and commentaries were excluded. Only the most recent peer-reviewed publication was included when multiple reports using the same study data existed. We limited our review to English language articles.^12^

### Study Selection and Data Extraction

Titles and abstracts of articles captured by literature searches were independently screened in duplicate by two reviewers (CP and CM). Articles flagged for full-text review by either reviewer were included in the full-text review, and the full-text review process was duplicated and independently completed by the same reviewers. Inter-rater reliability following full-text review was calculated using Cohens Kappa statistic and any disagreements on study inclusion were resolved via arbitration by a third reviewer (JJ).

### Quality Assessment

Data extraction and quality assessment for included studies were performed in duplicate (by CP and CM). An electronic data extraction template was developed, pilot tested, and refined prior to the initiation of data extraction. The extracted data elements included study design, sample size, study period, study setting, study population, patient type, study location, *Enterococcus* spp., VRE/VSE bacteremia definition, VRE therapy administered, and number of VRE and VSE bacteremia patients with the above stated outcome(s) of interest along with associated effect estimates and confidence intervals. Whenever required information was not reported, attempts were made to contact the first and/or corresponding authors to obtain missing information; after 2 attempts, authors were considered unresponsive. Data requests were limited to missing information on administered VRE treatment type(s), primary outcomes, and any secondary outcomes reported within the primary study.

Study quality was assessed using the Newcastle-Ottawa Scale (NOS) scale or Cochrane risk of bias tool. The NOS was used to establish quality of evidence within non-randomized cohort or case control studies, via a 9-star system.^13^ A study awarded a greater number of stars is considered to be of higher methodological study quality.^13^ Although we did not anticipate finding any RCTs, the Cochrane risk of bias tool was assigned to assess RCT study quality in the event an RCT meeting inclusion criteria was discovered.^10^

### Data Analysis

Outcome effect measures for each study were calculated using numbers of patients with VRE and VSE bacteremia with the outcome(s) of interest. We pooled studies of the same study design via inverse variance method and random effects modeling in Review Manager 5.3; summary effect measures are reported as odds ratios (OR) and 95% confidence intervals (CI) for mortality, and mean difference and standard deviation (SD) are reported for continuous LOS outcomes. When the median and interquartile ranges (IQR) were reported, the median was assumed to reflect the mean,^14^ and IQR was assumed to be 1.35 SD.^10^ Statistical heterogeneity was assessed using the I^2^ statistic, and VRE and VSE bacteremia outcomes were further explored via planned subgroup analyses of the following patient populations: (1) adult versus pediatric patients, (2) immunocompromised versus varied immune status patients, ICU versus non-ICU admissions, (3) multicenter versus single study site, and low versus moderate-to-high study quality for included cohort studies.^10^ Publication bias was examined via the visual interpretation of funnel plot symmetry and limited to the mortality outcomes.^10^

### Role of the Funding Source

The design, conduct, and reporting for this systematic review and meta-analysis was funded by the Ontario Agency for Health Protection and Promotion (Public Health Ontario).

## Results

### Literature Search

**Figure 1:**
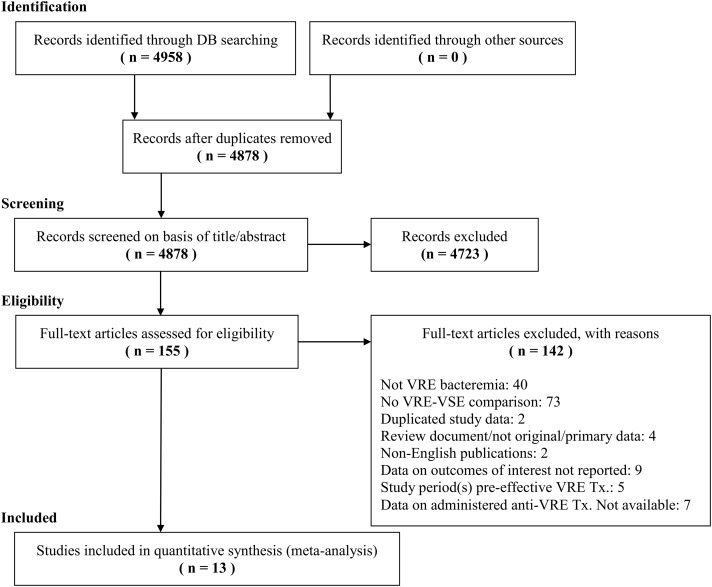
Preferred Reporting Items for Systematic Reviews and Meta-Analysis (PRISMA) flowchart of the literature search and study selection.

The literature searches identified 4,878 citations; among these, 155 citations were chosen for full-text review, and 20 studies were determined to meet our inclusion criteria (Figure 1). Of these, 1 study did not indicate a study period and 5 studies reported study periods between January 1997 and January 2000 and required confirmation of VRE therapies within each study. Corresponding authors were contacted, but administered VRE therapy information could not be obtained and all 7 studies were excluded from the review. Excluded study details are provided in Supplemental Table 3. Therefore, 13 studies were included in the systematic review.

### Description of Studies

The study characteristics of all included studies are outlined in Table 1. All were observational and retrospective studies, 12 of which were conducted between January 2000 and December 2011, following the formal regulatory approval of the first effective VRE therapy.^15^ ^–^ ^26^ The study by da Silva et al^27^ reported a study period between September 1998 and December 2008. However, these authors confirmed that all patients with VRE bacteremia were diagnosed after January 2000. Billington et al^16^ sampled all residents within a Canadian health zone who developed enterococcal bloodstream infections. We contacted these authors to obtain mortality and LOS information for study participants. In addition, 8 studies exclusively sampled adult patients within tertiary care hospital settings,^15^ ^,^ ^17^ ^–^ ^19^ ^,^ ^21^ ^,^ ^22^ ^,^ ^24^ ^,^ ^26^ and 4 of these studies were limited to immunocompromised patient populations^19^ ^,^ ^22^ ^,^ ^25^ ^,^ ^26^ such as hematopoietic stem cell transplant patients or chemotherapy recipients.

**Table 1:** Characteristics of Studies Included in Systematic Review and Meta-Analysis

				Sample Size, No.	
Study	Study Period	Location	Patient Population	VRE	VSE
**Cohort studies**					
Bar et al, 2006^15^	Nov 2000–Dec 2002	Richmond, VA USA	Adult	17	33
Billington et al, 2014^16^	2000–2008^a^	Calgary, Canada	Mixed	27	640
Butler et al, 2010^17^	Jan 2002–Dec 2003	St Louis, MO USA	Adult, Non-surgical, >2 days LOS	94	182
Cheah et al, 2013^18^	Jan 2002–March 2010	Victoria, Australia	Adult, >2 days LOS	116	116
Cho et al, 2013^19^	July 2009–Dec 2011	Seoul, Korea	Adult, neutropenia post CHEMO or SCT	24	67
da Silva et al, 2014^27^	Sep 1998–Dec 2008	Sao Jose do Rio Preto, Brazil	Mixed	30^c^	273^c^
Haas et al, 2010^20^	2001–2006^a^	Philadelphia, PA USA	Pediatrics	39	300
Marschall et al, 2013^21^	Jan 2006–Dec 2006	St. Louis, MO USA	Adult, CVC associated bacteremias	67	39
Mikulska et al, 2012^22^	2004–2011^a^	Genoa, Italy	Adult, allogeneic HSCT	9	58
Mohr et al, 2009^23^	Jan 2000–Dec 2009	58 sites, USA	Mixed,^b^ dap Tx.	151	211
Vydra et al, 2012^25^	Jan 2004–Dec 2008	Minneapolis, MN USA	Mixed, HSCT	50	43
Yoo et al, 2004^26^	Jan 2000–Dec 2001	Seoul, Korea	Adult, HSCT or cytotoxic CHEMO	19^D^	8
**Case control study**					
Peel et al, 2011^24^	Jan 2000–Dec 2009	Victoria, Australia	Adult	80	360

NOTE. LOS, length of stay; HSCT, hematopoietic stem cell transplantation; CHEMO, chemotherapy; dap Tx, daptomycin treatment; CVC, central venous catheter; SCT, stem cell transplantation.

a Months not reported.

b Assumed to be mixed, unconfirmed due to demographics being reported as ≤30 years of age.

c Data obtained by contacting study authors.

d A total of 8 VRE patients received VRE therapies and were included in the review.

All included studies defined patients with at least 1 VRE- or VSE-positive blood culture to be cases of bacteremia.^15^ ^–^ ^27^ Both *E. faecalis* and *E. faecium* were captured in 12 study samples,^15^ ^–^ ^25^ ^,^ ^27^ but the study by Yoo et al^26^ only included *E. faecium* infections. Outcome data for 2,575 bacteremias, specifically 1,863 VSE and 712 VRE bacteremia cases, were identified in our literature review.

### Outcomes

**Figure 2:**
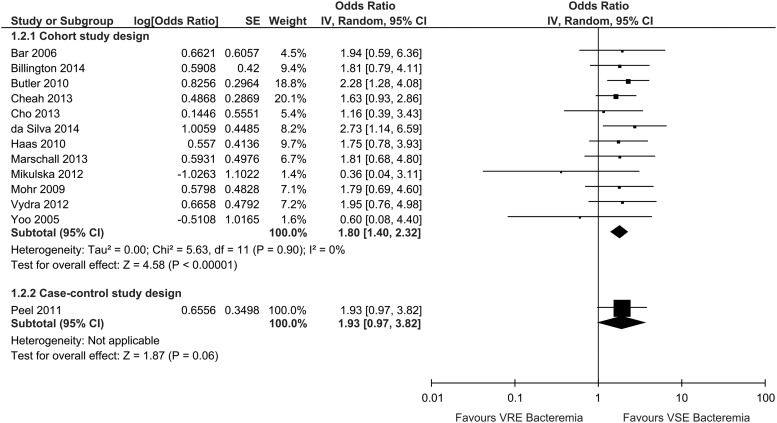
VRE and VSE bacteremia unadjusted in-hospital mortality risk by study design. Results of included studies for VRE and VSE bacteremia unadjusted in-hospital mortality risk stratified by study design. Abbreviations: 95% CI, 95% confidence interval; SE, standard error; IV, random, inverse-variance, random-effects method.

Of the reviewed studies, 12 studies were cohort studies and 1 was a case control study. When in-hospital mortality from the cohort studies were combined using unadjusted analysis, VRE bacteremia was associated with an increased risk of in-hospital death when compared to VSE bacteremia with no heterogeneity (OR, 1.80; 95% CI, 1.40–2.32; I^2^=0%; n=12) (Figure 2). The single case-control study did not report a statistically significant increase in risk of VRE bacteremia death when compared with VSE bacteremia in an unadjusted analysis (OR 1.93; 95% CI, 0.97–3.82)^24^; adjusted analyses were not reported.

Of the 12 cohort studies, 5 reported adjusted analyses for in-hospital mortality risk,^15^ ^,^ ^16^ ^,^ ^18^ ^,^ ^19^ ^,^ ^21^ and 2 studies found VRE bacteremia to be associated with adjusted mortality.^18^ ^,^ ^19^ Cheah et al^18^ adjusted for prior ICU admission, comorbidities measured by the Charlson Comorbidity Index, *Enterococcus* sp., additional non-enterococcal infections, time to effective therapy, and VRE bacteremia (OR, 1.21; 95% CI, 0.53–2.79]) via logistic regression analysis. Cho et al^19^ adjusted for severity of illness using Simplified Acute Physiology Index, length of hospitalization, and vancomycin resistance (hazard ratio [HR], 0.75; 95% CI, 0.24–2.36) via Cox proportional hazards modeling. VRE bacteremia was not included in the final models of the remaining 3 studies reporting adjusted mortality.^15^ ^,^ ^16^ ^,^ ^21^

The study by Cho et al was the only study to report on VRE/VSE bacteremia-attributable mortality, which was defined as death within 7 days of bacteremia when no other cause could be identified. There was no significant difference in attributable mortality risk between VRE and VSE bacteremia patients in the unadjusted analysis (6 of 24 patients with VRE bacteremia vs 15 of 67 patients with VSE bacteremia; OR, 1.15; 95% CI, 0.39–3.43).^19^

**Figure 3:**
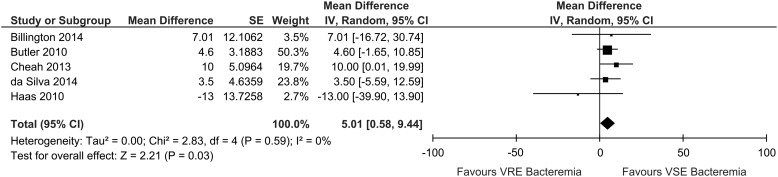
VRE and VSE bacteremia total hospital LOS mean difference. Results of studies reporting on VRE and VSE bacteremia total hospital LOS. Abbreviations: LOS, length of stay; 95% CI, 95% confidence interval; SE, standard error; IV, random, inverse-variance, random-effects method.

Total hospital LOS data were reported within 6 studies. Data reported by Butler et al^17^ and Cheah et al,^18^ and data obtained by contacting authors of Billington et al,^16^ da Silva et al,^27^ and Haas et al^20^ were pooled; VRE bacteremia was associated with a longer LOS than VSE bacteremia (mean difference, 5.01; 95% CI, 0.58–9.44; I^2^=0%; n=5) (Figure 3). Data from Cho et al were excluded because they defined LOS as the number of days from hospital admission to the development of clinically significant bacteremia, which is different from the LOS definition used in our review. Data from Yoo et al^26^ were excluded because their LOS estimates combined patients treated with effective and noneffective VRE therapy.

**Figure 4:**
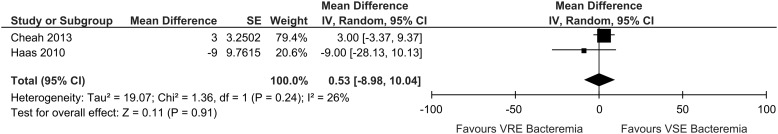
VRE and VSE post-bacteremia total hospital LOS mean difference. Results of studies reporting on VRE and VSE post-bacteremia hospital LOS. Abbreviations: LOS, length of stay; 95% CI, 95% confidence interval; SE, standard error; IV, random, inverse-variance, random-effects method.

Post-bacteremia LOS data reported by Cheah et al and Haas et al were also pooled via a meta-analysis. There was no significant difference in LOS following a VRE bacteremia compared with VSE bacteremia (mean difference, 0.53 [95% CI –8.98, 10.04]; I^2^=26%; n=2) (Figure 4). Yoo et al also reported on post-bacteremia LOS, but data were omitted because estimates combined patients treated with both effective and noneffective VRE therapy.

### Subgroup Analyses

**Figure 5:**
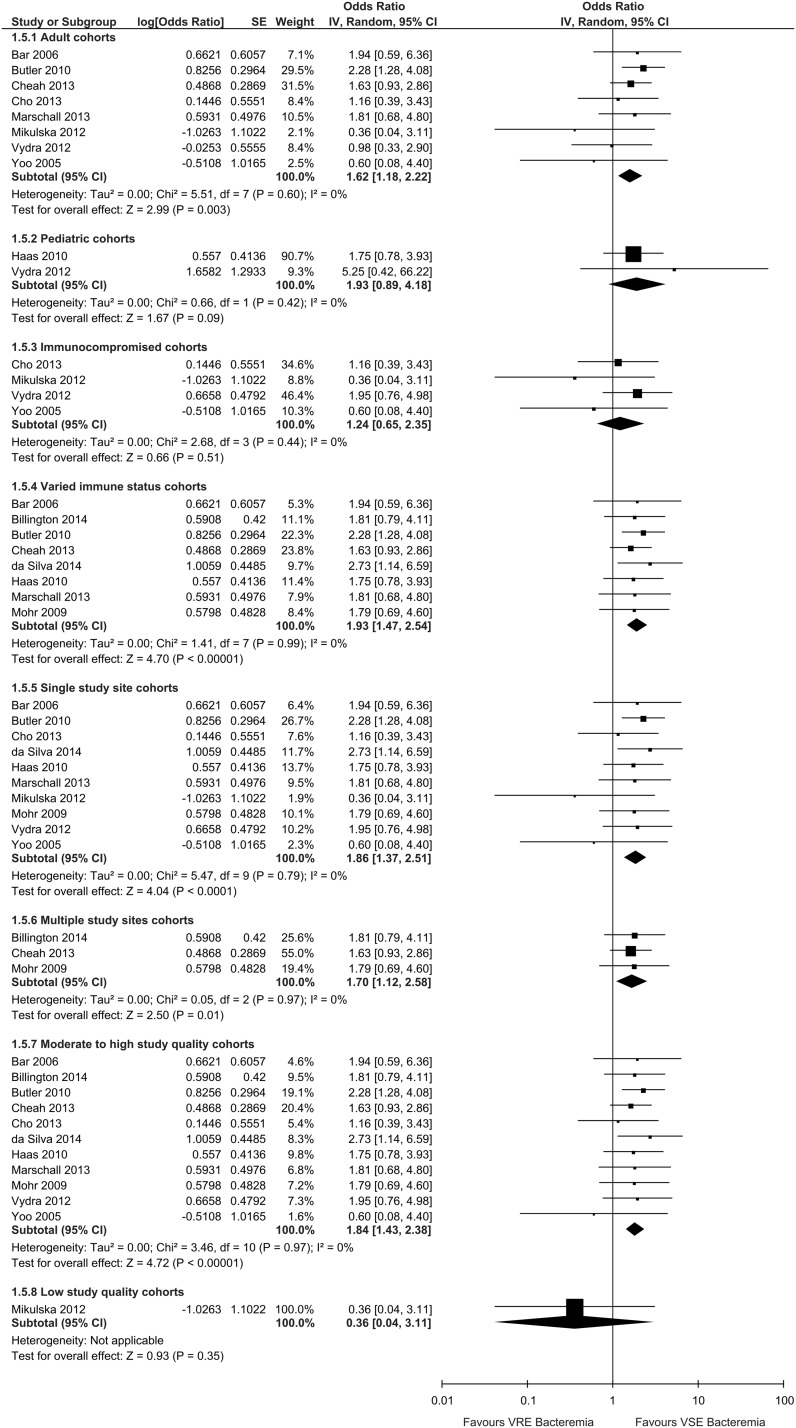
Subgroup analysis of VRE and VSE bacteremia un-adjusted in-hospital mortality risk by age, immune status, study site(s), and study quality, for each included cohort study reporting these data. Abbreviations: 95% CI, 95% confidence interval; SE, standard error; IV, random, inverse-variance, random-effects method.

No significant interactions were detected between any of the subgroups we had planned to analyze for in-hospital mortality including age (pediatric patients [OR, 1.62; 95% CI, 1.18–2.22] vs adult [OR, 1.93; 95% CI, 0.89–4.18]; interaction *P*=.68), immune status (immunocompromised patients [OR, 1.24; 95% CI, 0.65–2.35] vs varied immune status [OR, 1.93; 95% CI, 1.47–2.54]; interaction *P*=.21), study site (single center studies [OR, 1.85; 95% CI, 1.37–2.50] vs multicenter studies [OR, 1.70; 95% CI, 1.12–2.58]; interaction *P*=.75) and study quality (low-quality studies [OR, 0.36; 95% CI, 0.04–3.11] vs moderate- to high-quality studies [OR, 1.84; 95% CI, 1.43–2.37]; interaction *P*=.14) (Figure 5). The planned subgroup analysis for ICU stay was not performed due to unavailable data.

**Figure 6:**
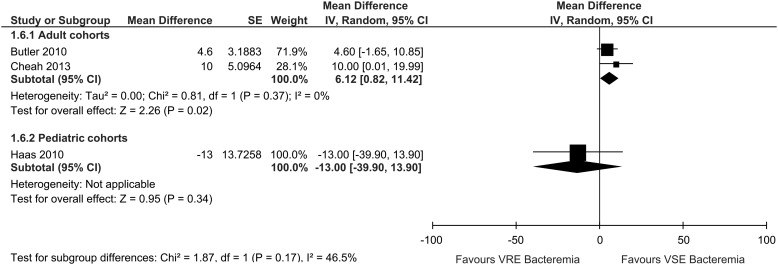
Subgroup analysis of VRE and VSE bacteremia hospital LOS by age, for each included cohort study reporting these data. Abbreviations: LOS, length of stay; 95% CI, 95% confidence interval; SE, standard error; IV, random, inverse-variance, random-effects method.

Age was not found to significantly influence total hospital LOS by subgroup analysis (pediatric patients [OR −13.00; 95% CI, −39.90–13.90] vs adult [OR, 6.12; 95% CI, 0.82–11.42]; interaction *P*=.17) (Figure 6). The remaining LOS subgroup analyses could not be performed due to a lack of studies in each companion subgroup. No significant interaction was detected in the subgroup analysis of post-bacteremia LOS by age (pediatric patients [OR, −9.0; 95% CI, −28.13–10.13] vs adult [OR, 3.0; 95% CI −3.37–9.37]; interaction *P*=.24) (Figure 4).

### Study Quality

Study quality ratings based on NOS criteria are presented in Table 2. Of the 13 studies reviewed, 12 were of moderate to high study quality, with the most frequent number of stars awarded per study being 6 or 7. Among all studies, patients with VRE and VSE bacteremia were selected from the same hospital population, and bacteremia diagnosis was confirmed by patient chart reviews or microbiology reports.

**Table 2:** Assessment of Study Quality, Based on the Newcastle-Ottawa Scale (NOS) Star System

Study	Selection	Comparability^a^	Outcome/Exposure	Total **S**tars
**Cohort studies**				
Bar et al, 2006^15^	****		***	7
Billington et al, 2014^16^	****		***	7
Butler et al, 2010^17^	****		***	7
Cheah et al, 2013^18^	****	**	***	9
Cho et al, 2013^19^	***	**	***	8
da Silva et al, 2014^27^	****		***	7
Haas et al, 2010^20^	***		***	6
Marschall et al, 2013^21^	***		***	6
Mikulska et al, 2012^22^	**		**	4
Mohr et al, 2009^23^	****		***	7
Vydra et al, 2012^25^	***		***	6
Yoo et al, 2004^26^	***		***	6
**Case control study**				
Peel et al, 2011^24^	***		***	6

a Illness severity and comorbid conditions were selected as the most important factors when assessing comparability.

### Publication Bias

**Figure 7:**
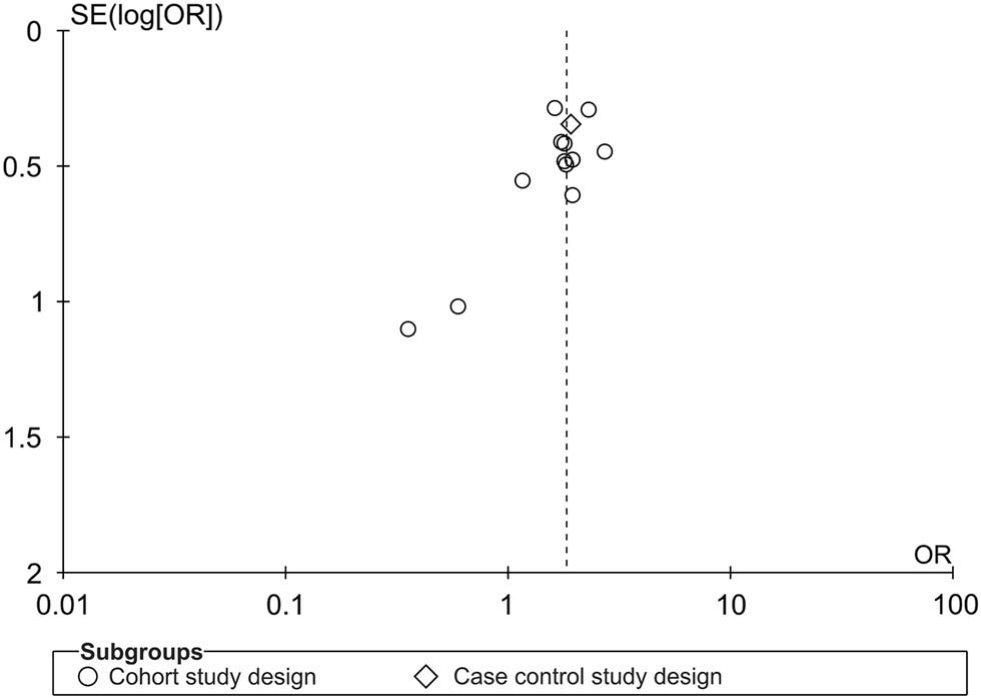
Asymmetrical funnel plot of VRE and VSE bacteremia in-hospital mortality effect estimates of all included studies.

The asymmetrical funnel plot indicates that the review’s in-hospital mortality estimates may be subject to publication bias (Figure 7).

## Discussion

In this systematic review and meta-analysis, we found that since the advent of effective VRE therapy, there remains an increased risk of in-hospital mortality associated with VRE bacteremia compared with VSE bacteremia. The mortality summary estimate demonstrated lack of heterogeneity across studies and no significant influence on the point estimate by age, immune status, study site(s), or study quality. VRE bacteremia was also associated with increased total hospital LOS and post-bacteremia LOS with no heterogeneity. The post-bacteremia LOS estimate was not statistically significant, which may have been due to lack of statistical power influenced by the small number of studies reporting on post bacteremia LOS outcomes.

Our finding, that there is an increased risk of mortality and LOS associated with VRE bacteremia when compared to VSE bacteremia, is consistent with 2 previous systematic reviews.^5^ ^,^ ^6^ In the systematic review by Salgado et al,^5^ the authors speculated that the increased morbidity and mortality could be because patients with VRE bacteremia were more likely to receive ineffective therapy.^5^ ^,^ ^6^ However, our findings suggest that a lack of effective therapy is not the explanation. It should be noted that our systematic review was unable to capture time to effective therapy. Thus, it is possible that patients with VSE bacteremia received effective therapy sooner than patients with VRE bacteremia because VRE may be less likely to be covered by empiric therapy, and effective therapy may only have been administered following a VRE-positive microbiological culture result.^6^ ^,^ ^15^ ^,^ ^18^ ^,^ ^19^

An alternative explanation for the observed increase in mortality risk and LOS could be differences in illness severity or comorbidities between patients with VRE and VSE bacteremia, particularly because patients with VRE bacteremia may have more comorbidities.^18^ ^,^ ^24^ ^,^ ^28^ Due to limited reporting of adjusted mortality and morbidity risks among included studies, we were unable to calculate adjusted summary estimates in this systematic review. Thus, the effect of confounding factors on our unadjusted mortality and LOS summary estimates remains unclear. We hypothesize that not adjusting for potential confounders (ie, comorbid conditions and severity of illness) may lead to overestimates of our associations of interest because patients colonized with VRE tend to have more comorbid conditions and more severe illness than patients not colonized with VRE.^18^

However, the earlier systematic review by DiazGranados et al,^6^ which only considered studies controlling for underlying severity of illness, found VRE bacteremia adjusted mortality risk to be greater in comparison to VSE bacteremia.

The worse outcomes associated with VRE bacteremia compared to VSE bacteremia may also be linked to differences in the causative species as there may have been proportionately more patients with *E. faecium* than *E. faecalis* in the VRE bacteremia group when compared to the VSE bacteremia group.^1^ ^,^ ^2^

Our results should be interpreted recognizing the systematic review’s limitations. First, studies included within each meta-analysis were non-randomized observational studies, and accordingly, our results reflect association and not causation. Second, as discussed above, only a small number of studies adjusted for potential confounders, and thus confounding may have influenced the investigated associations. Third, our results may be limited by the exclusive review of English language reports published after January 1997, but it is unlikely such language restrictions biased our findings.^12^ Fourth, the majority of studies sampled immunocompromised hospital patient populations, which limited our ability to generalize our findings to all healthcare settings. Last, our funnel plot suggests that there may be publication bias. However, the 2 studies that contributed to this asymmetry had high standard error and odds ratios close to 1. Thus, if the asymmetry in the funnel plot is due to publication bias, it would bias the results towards the null hypothesis.

We conclude that using the best available evidence, VRE bacteremia remains associated with increased risk of morbidity and mortality when compared with VSE bacteremia in the era of effective VRE therapy. Future research is needed to determine whether these results are related to unadjusted differences in the patient populations, differences in treatment effectiveness, or differences in proportions of patients with *E. faecalis* and *E. faecium* comprising the VRE and VSE bacteremias.

## Supplementary material

For supplementary material accompanying this paper visit http://dx.doi.org/10.1017/ice.2015.228. click here to view supplementary material
