# Supplementary material for: VRE and VSE Bacteremia Outcomes in the Era of Effective VRE Therapy: A Systematic Review and Meta-analysis
Source: Infect Control Hosp Epidemiol. 2015 Oct 5;37(1):26–35. doi: 10.1017/ice.2015.228 (PMC4707508; doi:10.1017/ice.2015.228)
Supplement: Supplementary file 1 [file S0899823X15002287sup001.docx]

Supplemental Table 1. Sample Medline search strategy

| 1 | (exp Enterococcus/ and (Bacteremia/ or exp Drug Resistance/ or exp Glycopeptides/ or Vancomycin/ or Vancomycin Resistance/)) or exp Enterococcus/de, ip or (Vancomycin/ and exp Drug Resistance/) or Vancomycin Resistance/ |
| --- | --- |
| 2 | ((bacteremia adj4 vancomycin$) or (drug resistan$ adj4 enterococc$) or (glycopeptide resistan$ adj4 enterococc$) or (vancomycin$ adj4 enterococc$) or (vancomycin$ adj4 resistan$) or vancomycin-resistan$ or VRE).mp. |
| 3 | "Cost of Illness"/ or Death Certificates/ or exp Epidemiologic Factors/ or exp Epidemiology/ or exp Morbidity/ or exp Mortality/ or "Outcome Assessment (Health Care)"/ or exp Risk/ or exp Treatment Outcome/ |
| 4 | (attributed or attributable or (burden adj2 (illness or disease$)) or comorbid$ or death or epidemiolog$ or incidence or morbid$ or mortality or outcome$ or prevalen$).mp. |
| 5 | (1 or 2) and (3 or 4) |
| 6 | limit 5 to (english language and yr="1997 -Current") |
| 7 | remove duplicates from 6 |

Supplemental Table 2. Grey-literature Search /Publications from ICP Websites/Conferences

| AMMI (Association of Medical Microbiology and Infectious Disease Canada)  - Website- no new references  - AMMI Conference (2011, 2012, 2013, 2014) - no new references  - AMMI Conference 2010 abstracts - no new references (obtained by JS contacting the organization) |
| --- |
| APIC (Association for Professionals in Infection Control and Epidemiology)  - Website - no new references   - - APIC Conference (2012 abstracts - no new references )   - APIC Conference 2009, 2010, and 2011 abstracts - no new references (obtained by JS contacting the organization)   - APIC Conference 2013 - not available |
| APSIC (Asia-Pacific Society of Infection Control)   - - Website - no new references |
| ICAR (International Conference on Anti-Microbial Research-2012 abstracts) - no new refs |
| IDSA (Infectious Disease Society of America)   - - Website - no new references   - ID Week (2012, 2013) - no new references |
| IFIC (International Federation for Infection Control)   - - Website - no new references   - IFIC conference 2011, 2012 and 2013 presentations - no new references   - IFIC conference 2009, 2010, 2011, 2012 abstracts - no new references (obtained by contacting the organization) |
| IPAC Canada (Infection prevention and control Canada)   - - Website - no new references   - IPAC Canada/CHICA conference 2013 - no new references   - IPAC Canada/CHICA conference 2009, 2010, 2011 and 2012 - no new references |
| IPS (Infection Prevention Society)   - - Website- no new references   - IPS conference (2010 & 2011 Presentation schedule available on-line) - no new references |
| HIS (Healthcare Infection Society)   - - Website- no new references   - HIS conference only 2012 - no new refs (obtained by contacting the organization) |
| SHEA (Society for healthcare epidemiology of America)   - - Website - no new references   - SHEA Conference 2009-2011 - cannot access conference publications on-line |

All searches were run on January 2015 with VRE and Vancomycin keyword searches, by CP

Supplemental Table 3. Excluded studies with exclusion reasons

| Author, Year | Reasons for Study Exclusion |
| --- | --- |
|  |  |
| Bhavnani, 2000 | Reports the use of effective VRE therapy among 16 VRE bacteremia patients within the study sample, but does not specify mortality outcomes for these cases. Study authors were contacted, but the requested data was not obtained. Study period, 1995-1997 |
| DiazGranados, 2005 | Reports the use of effective VRE therapy among 8 VRE bacteremia patients within the study sample, but does not specify the mortality outcomes for these cases. Study authors were contacted, but the requested data was not obtained. Study period, November 1994 to January 2001. |
| Garbutt, 2000 | Reports the use of effective VRE therapy among 20 VRE bacteremia patients within the study sample, but does not specify the mortality outcomes for these cases. Study authors were contacted, but the requested data was not obtained. Study period, January 1995 and April 1997. |
| Ibrahim, 2000 | Reports the use of effective VRE therapy, but does not specify the administered VRE therapy type(s), or the mortality outcomes of VRE bacteremia cases administered effective VRE therapy. Study authors were contacted, but the requested data was not obtained. Study period, July 1997 and July 1999. |
| Krcmery, 2001 | The study is cited within a published letter to the editor of a peer reviewed journal, but the exact study period was not reported. Therefore it could not establish whether the study was conducted pre or post 1997, a selection criteria for establishing effective VRE therapy within this review. Study authors were contacted, but the requested data was not obtained. Study period not reported |
| Lodise, 2002 | Reports the use of effective VRE therapy in a single VRE bacteremia patient within the study sample, but does not specify the mortality outcomes for this case. Study authors were contacted, but the requested data was not obtained. Study period, 1996-2000. |
| Vergis, 2001 | Repots the use of effective VRE therapy in 6 VRE bacteremia patients, who later died. The publication does not specify the administered VRE therapy type, which included medications considered effective and non-effective within the current review. Study authors were contacted, but the requested data was not obtained. Study period, February 1995 and March 1999. |
